# Predictors of specialist care referrals (SCR) following emergency department review or hospital admission in adults with previous acute COVID-19: a prospective UK cohort study

**DOI:** 10.1186/s12873-024-01164-x

**Published:** 2025-01-23

**Authors:** Anita Saigal, Songyuan Xiao, Owais Siddique, Prasheena Naran, Heba M Bintalib, Camila Nagoda Niklewicz, George Seligmann, Sindhu Bhaarrati Naidu, Amar J Shah, Chibueze Ogbonnaya, John R Hurst, Marc Ci Lipman, Swapna Mandal

**Affiliations:** 1 UCL Respiratory, University College London, London, UK; 2 Respiratory Medicine, Royal Free London NHS Foundation Trust, London, UK; 3 Department of Respiratory Care, King Saud Bin Abdulaziz University for Health Sciences, Jeddah, Saudi Arabia; 4 King Abdullah International Medical Research Centre, Jeddah, Saudi Arabia; 5 Institute of Child Health, University College London, London, UK

**Keywords:** Long-COVID, Ongoing symptomatic COVID-19, Emergency department, SARS-CoV-2

## Abstract

**Background:**

Long-COVID research to date focuses on outcomes in non-hospitalised vs. hospitalised survivors. However Emergency Department attendees (post-ED) presenting with acute COVID-19 may experience less supported recovery compared to people admitted and discharged from hospital (post-hospitalised group, PH).

**Objective:**

We evaluated outcomes and predictors of specialty care referrals (SCR) in those with ongoing symptomatic Long-COVID, comparing post-ED and PH adults.

**Methods:**

This prospective observational cohort study evaluates 800 PH and 484 post-ED adults from a single hospital in London, United Kingdom. Participants had either confirmed laboratory-positive SARS-CoV-2 infection or clinically suspected acute COVID-19 and were offered post-COVID clinical follow-up at approximately six weeks after their ED attendance or inpatient discharge, to assess ongoing symptoms and support recovery. Multiple logistic regression determined associations with specialist care referrals (SCR) to respiratory, cardiology, physiotherapy (including chest physiotherapy), and mental health services.

**Results:**

Presence of at least one Long-COVID symptom was lower in adults attending ED services with acute COVID-19 compared to those hospitalised (70.1% post-ED vs. 79.5% PH adults, *p* < 0.001). Total number of Long-COVID symptoms was associated with increased SCR in all patients (adjusted odds ratio (aOR) = 1.26, 95%CI:1.16, 1.36, *p* < 0.001), with post-ED adults more likely to need a SCR overall (aOR = 1.82, 95%CI:1.19, 2.79, *p* = 0.006). Post-ED adults had higher SCR to both physiotherapy (aOR = 2.59, 95%CI:1.35, 4.96, *p* = 0.004) and mental health services (aOR = 3.84, 95%CI:2.00, 7.37, *p* < 0.001), with pre-existing mental illness linked to the latter (aOR = 4.08, 95%CI:1.07, 15.6, *p* = 0.04).

**Conclusions:**

We demonstrate greater specialist care referrals to mental health and physiotherapy services in patients attending the ED and discharged with acute COVID-19, compared to those admitted, despite lower ongoing COVID-19 symptom burden. Total number of symptoms, pre-existing co-morbidity such as smoking status, cardiac co-morbidities, and mental health illnesses may predict those requiring healthcare input. This information may enable better post-COVID support for ED attendees, a distinct group who should not be neglected when preparing for future pandemics.

**Trial registration:**

This study had HRA approval (20/HRA/4928).

**Supplementary Information:**

The online version contains supplementary material available at 10.1186/s12873-024-01164-x.

## Introduction

### Background

The United Kingdom National Institute for Health and Care Excellence (NICE) defines Long-COVID as symptoms persisting for greater than four weeks following acute SARS-CoV-2 infection [1]. This research focuses on the subset within this umbrella defined as having ‘ongoing symptomatic COVID-19’ between 4 and 12 weeks rather than those experiencing ‘post-COVID-19 syndrome’ at beyond twelve weeks. This is due to the need to identify individuals at risk of Long-COVID who could be directed to therapeutic support from healthcare services earlier.

Patients attending an emergency department with acute COVID-19 symptoms and clinical signs of pneumonia, despite being discharged within twenty-four hours, represent individuals with moderate acute COVID-19, in keeping with the World Health Organisation definition of the severity of acute illness [2]. They represent a unique group of emergency attendees with acute COVID-19 infection.

Two UK studies [3, 4] have evaluated outcomes in this cohort of post-ED adults and have demonstrated a greater Long-COVID symptom burden and lower self-reported health status [3], in comparison to hospitalised individuals (PH: post-hospitalised). Prior research identified that up to 44% of post-ED adults (*n* = 199) need an in-person review at four weeks [4] with the first [3] study highlighting a similar number of medical specialist care referrals (SCR) across both groups (18.9% (*n* = 88) in post-ED adults vs. 16.1% (*n* = 234) in PH adults). This study evaluated predictors of poor functional recovery [3], but not of specialty care referrals. Systematic reviews highlight the latter predictor to be of importance within care models for COVID-19 [5] and qualitative evaluation [6] highlights individuals with long-COVID perceive themselves as eligible for SCR, suggesting this likely correlates with the healthcare resource required to address recovery in these individuals.

### Importance

A systematic review [7] to date has evaluated Long-COVID outcomes in those hospitalised compared to those non-hospitalised (*n* = 122 hospitalised, 18 non-hospitalised, 54 mixed studies). This has shown a greater presence of a Long-COVID symptom in adults hospitalised (*n* = 48 studies; 52.6%) compared to 34.5% in non-hospitalised adults (*n* = 11 studies). The accuracy of this symptom burden may be limited due to recall bias in non-hospitalised adults who have either self-diagnosed acute COVID-19 or reported their ongoing Long-COVID symptoms rather than being objectively assessed. Furthermore, the distinct intermediary group of post-ED adults has not been incorporated into this systematic research evaluation and future service planning therefore cannot account for these distinct groups accurately.

Hence, this study intended to both compare early Long-COVID symptom burden and predictors of SCR in those with ongoing symptomatic Long-COVID, comparing post-ED and PH adults with acute COVID-19. This study additionally analysed two pandemic timepoints to review if any changes in patterns of SCR arose as COVID-19 treatment evolved over time.

## Methods

### Study design

We conducted a single-centre prospective observational cohort study. This study had UK Health Research Authority (HRA) and Health and Care Research Wales approval (HRA number 20/HRA/4928). All participants consented to participate in the study at their initial follow-up consultation.

### Study setting

A virtual post-COVID service was established at the Royal Free London NHS Foundation Trust (methodology previously reported [8, 9]). We completed a virtual clinical review between six to twelve weeks following ED hospital attendance or discharge after an acute inpatient stay (see information on eligibility below in participant selection). Patients with abnormal blood tests and/or chest radiograph findings at discharge were invited to have tests repeated. We asked participants if they had any of the fourteen Long-COVID physical symptoms (determined by the North Central London ‘Assessing Recovery from COVID-19’ (ARC) consortium [8]) and psychological symptoms using the Patient Health Questionnaire-2 score (PHQ-2) [10] and Trauma Screening Questionnaire score (TSQ) [11]. Subjective breathlessness, cough, fatigue, and sleep quality were assessed on an eleven-point Likert scale from 0 to 10 (where 0 represented ‘I do not have this problem’ and 10 represented ‘this symptom is very significant’) and current breathlessness was assessed further using the Medical Research Council scale [12]. Lastly, the British Society of Thoracic Imaging (BSTI) classification [13] was used for coding chest radiographs, and blood biomarkers were measured using standard laboratory analysers.

Patients were referred onward for a specialty care referral (SCR) following a virtual review (phone call) and were referred according to protocolised pathways as displayed in Supplementary Figs. 2, 3 and 4. Participants were directed towards both a respiratory clinic and for physiotherapy and physical rehabilitation resources, if they had persistent disabling respiratory symptoms of breathlessness or cough with little improvement compared to the point of discharge, determined by their VAS breathlessness score. This therefore represented patients with symptomatic breathlessness or dysfunctional breathing suggestive of a breathing pattern disorder attributable to COVID-19 infection. Patients identified to have persistent depressive (PHQ-2 score ≥ 2) or post-traumatic stress symptoms (TSQ score ≥ 6) were encouraged to self-refer to Improving Access To Psychological (IAPT) services but were supported with this if unable to do this themselves.

Participants were directed onward to cardiology clinics if they had ongoing chest pain, persistently abnormal blood tests including raised troponins or N-terminal pro-B-type natriuretic peptide (NT-proBNP) above their baseline blood tests which had been performed during their hospital admission or ED visit (regardless of whether they had existing cardiac disease). This process was intended to select patients with cardiological symptoms that had arisen secondary to acute COVID-19 infection. Additionally, referrals were made to neurology for concerns with memory impairment or “brain fog” where thought to be a result of acute COVID-19, and finally chronic fatigue services, where fatigue was the predominant persisting symptom.

### Participant selection

#### Inclusion criteria

An attempt was made to contact every patient aged ≥ 18-years-old who had either been (i) discharged from our emergency department (post-ED) with radiographic changes of COVID-19 pneumonia or (ii) hospitalised with acute COVID-19 pneumonia (with or without a confirmatory SARS-CoV-2 positive swab) and discharged.

All participants provided verbal consent at their initial follow-up consultation and data were de-identified prior to analysis. We then identified participants who had been admitted and discharged between the two peak admission periods of the 29th February – 5th April 2020 (Wave 1) and 10th December 2020–8th February 2021 (Wave 2). These two timepoints were used to determine if SCR differed with use of new non-pharmaceutical (contact tracing) and pharmaceutical interventions (vaccine roll-out [14], biological therapies, anti-viral therapies, and steroid treatment [15]). Admission criteria for inpatient hospitalisation remained the same between the two waves.

For the purposes of analysis, we selected the first 400 PH adults who had been consecutively admitted and subsequently discharged from our inpatient wards during both the 29th February – 5th April 2020 (Wave 1) and the 10th December 2020–8th February 2021 (Wave 2). The total identified PH cohort for this analysis was 800 individuals and this represented an overall of 54% of ward admissions during these periods.

During the same established timepoints, we identified a total of 484 eligible post-ED patients across both Wave 1 and 2, who were admitted with acute COVID-19 symptoms and radiological evidence of COVID-19 pneumonia and had subsequently been discharged within twenty-four hours. This represented all ED admissions with radiological evidence of acute COVID-19 pneumonia during the same corresponding time periods as documented above.

#### Exclusion criteria

We excluded patients from follow-up and analysis if they were unable to participate in virtual follow-up call due to dementia, frailty or hospital-acquired COVID-19 (defined as a positive SARS-CoV-2 swab 7–14 days following admission). Participants were excluded if they had been admitted to another hospital and subsequently transferred to our inpatient wards and seen under our follow-up service.

**Fig. 1:**
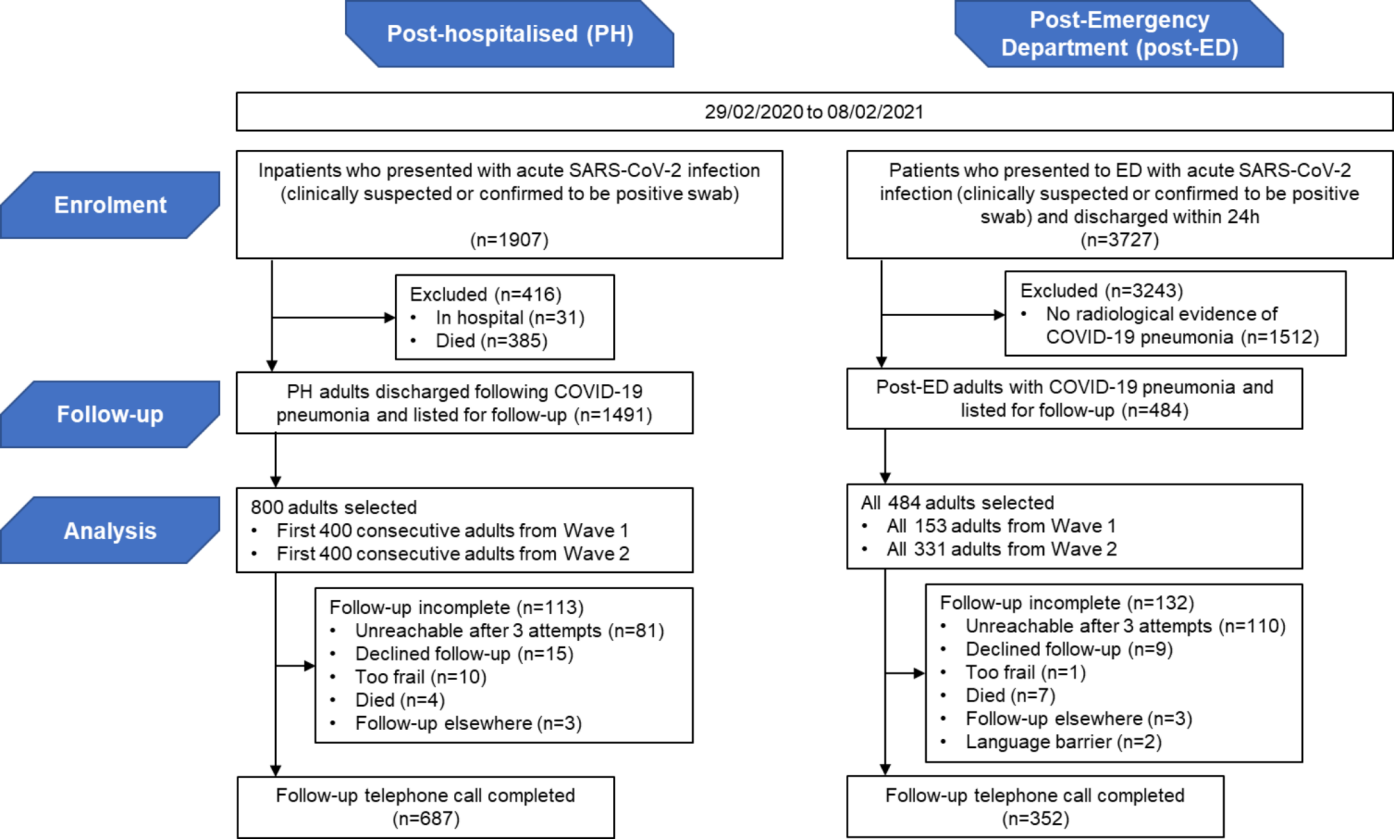
Study participants in post-hospitalised (PH) and post-emergency department (post-ED) groups

Figure 1 shows the enrolment and numbers achieved at follow-up, and Supplementary Fig. 1 details the number of patients enrolled across Waves 1 and 2. This study is reported in line with the strengthening the reporting of observational studies in epidemiology (STROBE) statement [16].

### Sample size

To determine the required sample size for a power of 80% at 5% level of significance, we used data from Heightman et al. [3] to provide estimates for the percentage of referrals to any of the four main specialties and for both ward and post-ED patients. Based on these estimates we computed the total sample size required for the main statistical analysis (logistic regression) with 80% power to be a total sample size of 598 participants, where the other predictors in the model increase the pseudo r-squared by 10%. This total sample size is for both for the post-ED and ward patients assuming an equal split in the sample size across both groups.

### Outcomes and analysis

#### Symptom burden

Descriptive outcomes include (a) the presence of Long-COVID which was defined as the presence of at least one of fourteen symptoms (listed in Table 2) and (b) the total number of Long-COVID symptoms across both post-ED and PH adults. We also report the total number of SCR to cardiology, chronic fatigue, memory clinic, mental health, neurology, physiotherapy and respiratory services. Subgroup analysis (Supplementary Table 2) analysed SCR patterns between Wave 1 and 2 participants.

We used IBM SPSS statistics for Windows (Version 28) to analyse the data. We tested descriptive data (displayed in Tables 1 and 2) for normality. Normally and non-normally distributed continuous data were summarised using mean and standard deviation (SD) and median and interquartile range (IQR) respectively and compared between PH and post-ED groups using two-tailed Student’s t-tests and Mann-Whitney-U tests respectively. Frequencies and percentages are presented for categorical variables and chi-squared tests compared PH and post-ED groups. A p-value of < 0.05 was considered statistically significant.

**Table 1:** Baseline patient demographics and co-morbidities at admission for PH and post-ED adults

Variable*	PH adults *N* = 800	Post-ED adults *N* = 484	*p*-value
**Patient characteristics**			
Age (years)	61 (51–74)	51 (41–63)	**< 0.001**
Female Gender	39.6% *N* = 317/800	47.1% *N* = 172/484	**0.009**
Ethnic minorities	48.8% *N* = 376/771	61.6% *N* = 297/482	**< 0.001**
Never Smokers	64.8% *N* = 504/778	60.9% *N* = 260/427	0.18
BMI (kg/m^2^)	27.2 (24.2–30.1) *N* = 540	27.4 (24.3–30.9) *N* = 305	0.56
Clinical Frailty Scale (using the Rockwood Frailty Scale)	3 (2–4) *N* = 716	2 (2–3) *N* = 439	**< 0.001**
**Pre-existing co-morbidities**			
Cardiac Disease (any)	17.8% *N* = 142/799	11.0% *N* = 53/482	**0.001**
Cerebrovascular Disease	7.3% *N* = 57/786	2.6% *N* = 12/456	**0.001**
Chronic Kidney Disease	13.6% *N* = 107/788	3.2% *N* = 15/472	**< 0.001**
Diabetes Mellitus	27.2% *N* = 210/772	13.8% *N* = 66/480	**< 0.001**
Hypertension	44.5% *N* = 350/786	25.3% *N* = 121/478	**< 0.001**
Immunocompromised	9.5% *N* = 76/800	3.5% *N* = 17/482	**< 0.001**
Lung Disease (any)	18.9% *N* = 151/800	17.0% *N* = 82/482	0.40
Mental health Disorder (any)	13.3% *N* = 106/800	6.0% *N* = 29/482	**< 0.001**
**Hospital Attendance Data**			
Total number of symptoms at onset of hospital attendance	3 (2–4)	2 (1–4)	**< 0.001**
NEWS2 score at onset of hospital attendance	4 (2–6) *N* = 751	2 (1–4) *N* = 448	**< 0.001**
Positive COVID swab	90.4% *N* = 723/800	67.5% *N* = 285/422	**< 0.001**
Pulmonary embolus	6.1% *N* = 46/756	1.1% *N* = 5/451	**< 0.001**

BMI: Body-mass index; NEWS2: National Early Warning Score 2

*All continuous data were non-normally distributed and presented as median (IQR), while categorical data were presented as number (N) and percentage (%). Mann-Whitney-U test was used to compare non-normally distributed continuous data. Chi-squared test was used to compare categorical data

**Table 2:** Clinical outcomes at initial follow-up consultation for post-ED and PH adults

Variable*	PH adults *N* = 687	Post-ED adults *N* = 352	*p*-value
Time from hospital discharge/ED attendance to follow-up appointment (days)	61 (52–83) *N* = 677	75 (55–106) *N* = 347	**< 0.001**
Time from onset of symptoms to time of follow-up appointment (days)	69 (59–92) *N* = 604	85 (66–112) *N* = 304	**< 0.001**
**Long-COVID symptom burden at follow-up**			
Abdominal pain	5.1% *N* = 34/671	5.5% 19/346	0.77
Anorexia	5.1% *N* = 34/671	4.0% *N* = 14/346	0.47
Anosmia	8.8% *N* = 59/671	6.6% *N* = 23/346	0.23
Breathlessness	16.6% *N* = 104/628	13.7% *N* = 6/336	0.24
Chest pain	7.6% *N* = 51/672	9.0% *N* = 31/346	0.45
Chest tightness	11.2% *N* = 75/671	11.6% *N* = 40/346	0.86
Confusion	13.0% *N* = 87/671	9.6% *N* = 33/345	0.11
Cough	22.2% *N* = 138/623	17.3% *N* = 58/336	0.07
Diarrhoea	4.2% *N* = 28/671	4.0% *N* = 14/346	0.92
Fatigue	11.9% *N* = 74/622	8.0% *N* = 27/337	0.06
Focal weakness	10.1% *N* = 68/672	7.5% *N* = 26/346	0.17
Myalgia	19.1% *N* = 128/671	17.1% *N* = 59/345	0.44
Peripheral oedema	10.0% *N* = 67/671	4.3% *N* = 15/346	**0.002**
Sleep quality decrease	29.2% *N* = 179/612	28.2% *N* = 93/330	0.73
Any 1 of 14 Long-COVID symptoms at follow-up	79.5% *N* = 534/672	70.1% *N* = 242/345	**< 0.001**
Total number of symptoms at follow-up	2 (1–4) *N* = 675	2 (0–4) *N* = 346	**< 0.001**
**Mental health burden**			
Depressive symptoms highlighted by a Patient Health Questionnaire-2 (PHQ-2) score ≥ 2	19.9% *N* = 129/647	19.1% *N* = 65/341	0.74
Post-traumatic stress symptoms highlighted by a Trauma Screening Questionnaire (TSQ) score ≥ 6	6.3% *N* = 38/604	3.5% *N* = 12/345	0.06
**Functional recovery**			
Patients reporting feeling back to normal	60.9% *N* = 271/445	74.7% *N* = 163/218	**0.001**
Patients reporting recovery as 0-100 on VAS scale (with 100 reflecting best recovery)	90 (70–99) *N* = 614	90 (75–100) *N* = 330	**0.006**
Number of patients back to work:			
Yes	29.4% *N* = 190/647	46.1% *N* = 155/336	**< 0.001**
No	23.8% *N* = 154/647	20.5% *N* = 69/336	
Not applicable	46.8% *N* = 303/647	33.3% *N* = 112/336	
**Radiology**			
Chest X-Ray result:			
Normal	67.9% *N* = 399/588	79.4% *N* = 201/253	**< 0.001**
Significantly improved (PCVCX1)	20.4% *N* = 120/588	15.8% *N* = 40/253	
**Specialty Care Referral (SCR)**			
Any 1 of 4 Referrals (respiratory/cardiology/physiotherapy/mental health)	25.0% *N* = 156/625	32.6% *N* = 114/350	**0.011**
Respiratory Referrals	10.2% *N* = 63/619	8.9% *N* = 31/350	0.51
Cardiology Referrals	10.9% *N* = 68/626	9.1% *N* = 32/351	0.39
Physiotherapy Referrals for exercise rehabilitation and/or management of dysfunctional breathing	7.5% *N* = 41/626	14.2% *N* = 50/351	**0.001**
Mental Health referrals	9.0% *N* = 56/625	15.1% *N* = 53/351	**0.003**
Neurology Referrals	0.2% *N* = 1/626	0.0% *N* = 0/351	0.46
Referrals To Chronic Fatigue Services	0.3% *N* = 2/626	0.0% *N* = 0/351	0.30
Memory Clinic Referrals	0.5% *N* = 3/626	0.0% *N* = 0/351	0.20

*All continuous data were non-normally distributed and presented as median (IQR), while categorical data were presented as number (N) and percentage (%). Mann-Whitney-U test was used to compare non-normally distributed continuous data. Chi-squared test was used to compare categorical data

#### Predictors of SCRs

We evaluated associations with the four most prevalent SCRs individually (respiratory, cardiology, physiotherapy, mental health services), and associations with any of these four referrals. Univariable (unadjusted) and multiple (adjusted) logistic regression analysis was conducted firstly in the entire cohort (Table 3 and Supplementary Table 3) and then specifically in post-ED adults alone (Table 4 and Supplementary Table 4). The duration between COVID-19 symptom onset and the follow-up appointment was adjusted for.

**Table 3:** Multiple logistic regression model for SCR in all participants

	Respiratory Referrals (*N* = 614)		Cardiology Referrals (*N* = 619)		Physiotherapy Referrals (*N* = 619)		Mental health Referrals (*N* = 618)		Any 1 of 4 Referrals (*N* = 614)	
Predictor Variable*	OR (95% CI)	*p*-value	OR (95% CI)	*p*-value	OR (95% CI)	*p*-value	OR (95% CI)	*p*-value	OR (95% CI)	*p*-value
**Long-COVID symptoms**										
Number of Long-COVID symptoms (at follow-up)	1.27 (1.13, 1.41)	**< 0.001**	1.13 (1.00, 1.27)	0.05	1.23 (1.09, 1.38)	**< 0.001**	1.34 (1.19, 1.50)	**< 0.001**	1.26 (1.16, 1.36)	**< 0.001**
**Patient Demographics**										
Post-ED	0.82 (0.43, 1.58)	0.56	1.39 (0.73, 2.65)	0.32	2.59 (1.35, 4.96)	**0.004**	3.84 (2.00, 7.37)	**< 0.001**	1.82 (1.19, 2.79)	**0.006**
Age (years)	1.00 (0.98, 1.03)	0.82	1.03 (1.00, 1.06)	**0.023**	1.02 (0.99, 1.04)	0.22	1.01 (0.98, 1.03)	0.72	1.02 (1.00, 1.03)	0.06
Sex = Male	1.52 (0.82, 2.82)	0.18	1.03 (0.56, 1.88)	0.93	0.81 (0.45, 1.45)	0.48	0.67 (0.37, 1.21)	0.18	1.04 (0.70, 1.54)	0.84
Ethnicity = Ethnic minorities	0.72 (0.40, 1.30)	0.27	0.91 (0.49, 1.66)	0.74	1.28 (0.70, 2.33)	0.42	1.09 (0.59, 2.01)	0.78	0.93 (0.62, 1.37)	0.70
Ever smoked	0.77 (0.43, 1.38)	0.38	1.27 (0.71, 2.30)	0.42	0.97 (0.53, 1.75)	0.91	2.04 (1.11, 3.74)	**0.022**	1.09 (0.94, 1.60)	0.68
BMI (kg/m^2^)	1.02 (0.96, 1.07)	0.59	1.03 (0.98, 1.09)	0.28	1.07 (1.02, 1.13)	**0.010**	1.00 (0.94, 1.05)	0.85	1.00 (0.96, 1.04)	0.89
CFS on admission	1.05 (0.80, 1.39)	0.71	0.86 (0.65, 1.15)	0.32	1.17 (0.88, 1.54)	0.28	0.85 (0.63, 1.16)	0.32	0.91 (0.76, 1.09)	0.31
**Patient Co-morbidities**										
Cardiac disease (any)	0.86 (0.26, 2.88)	0.81	1.52 (0.56, 4.15)	0.41	0.78 (0.25, 2.41)	0.66	2.59 (1.00, 6.67)	0.05	1.19 (0.59, 2.14)	0.63
Cerebrovascular disease	0.30 (0.03, 2.84)	0.29	1.45 (0.36, 5.73)	0.60	0.33 (0.06, 1.92)	0.22	1.65 (0.33, 8.23)	0.54	1.24 (0.44, 3.50)	0.69
Chronic kidney disease	0.57 (0.12, 2.64)	0.47	0.29 (0.06, 1.35)	0.11	0.26 (0.06, 1.23)	0.09	0.76 (0.16, 3.56)	0.72	0.65 (0.26, 1.64)	0.36
Diabetes	1.08 (0.37, 3.15)	0.89	0.82 (0.31, 2.18)	0.69	0.30 (0.10, 0.89)	**0.031**	1.01 (0.36, 2.85)	0.99	0.76 (0.38, 1.52)	0.44
Hypertension	0.79 (0.28, 2.27)	0.66	0.72 (0.29, 1.83)	0.49	0.55 (0.22, 1.41)	0.21	1.29 (0.51, 3.28)	0.59	0.86 (0.46, 1.64)	0.65
Immunosuppressed	0.74 (0.17, 3.28)	0.69	1.25 (0.37, 4.17)	0.72	0.59 (0.14, 2.42)	0.46	0.55 (0.13, 2.29)	0.41	0.86 (0.36, 2.03)	0.72
Lung condition (any)	0.58 (0.17, 1.91)	0.37	0.59 (0.20, 1.75)	0.34	0.66 (0.24, 1.81)	0.42	0.82 (0.28, 2.43)	0.73	0.70 (0.34, 1.45)	0.34
Mental health condition (any)	0.33 (0.08, 1.41)	0.14	0.43 (0.12, 1.54)	0.20	0.65 (0.21, 2.02)	0.46	2.49 (0.91, 6.81)	0.08	1.05 (0.49, 2.24)	0.91
Number of pre-existing morbidities	1.18 (0.54, 2.57)	0.69	1.30 (0.70, 2.42)	0.41	1.65 (0.88, 3.08)	0.12	0.89 (0.48, 1.62)	0.69	1.15 (0.73, 1.80)	0.55
**Patient Admission data**										
Number of acute COVID-19 symptoms	0.88 (0.74, 1.04)	0.14	0.94 (0.79, 1.12)	0.47	0.75 (0.62, 0.90)	**0.002**	0.94 (0.80, 1.12)	0.51	0.87 (0.77, 0.97)	**0.018**
Symptom onset to follow-up date (days)	1.00 (0.99, 1.01)	0.68	0.99 (0.98, 1.00)	0.06	1.00 (0.99, 1.01)	0.47	0.99 (0.98, 1.00)	0.10	0.99 (0.99, 1.00)	0.06

BMI: Body mass index; CFS: Clinical frailty scale (using the Rockwood Frailty Scale).*Table data are presented as adjusted odds ratio (aOR) and its 95% confidence ratio (95%CI)

**Table 4:** Multiple logistic regression model for SCR in post-ED adults

Referral Outcome	Respiratory (*N* = 254)		Cardiology (*N* = 254)		Physiotherapy (*N* = 254)		Mental health (*N* = 254)		Any 1 of 4 referrals (*N* = 254)	
Predictor Variable*	OR (95% CI)	*p*-value	OR (95% CI)	*p*-value	OR (95% CI)	*p*-value	OR (95% CI)	*p*-value	OR (95% CI)	Wald test *p*-value
**Long-COVID symptoms**										
Number of Long-COVID symptoms (at follow-up)	1.43 (1.15, 1.77)	**0.001**	1.37 (1.09, 1.70)	**0.006**	1.35 (1.12, 1.62)	**0.002**	1.39 (1.17, 1.64)	**< 0.001**	1.42 (1.23, 1.65)	**< 0.001**
**Patient characteristics**										
Age (years)	1.02 (0.97, 1.07)	0.45	1.05 (1.00, 1.10)	0.06	1.00 (0.97, 1.04)	0.91	0.99 (0.96, 1.02)	0.50	1.01 (0.99, 1.04)	0.40
Sex = Male	1.38 (0.48, 3.98)	0.55	1.47 (0.49, 4.40)	0.49	0.36 (0.15, 0.85)	**0.021**	0.64 (0.28, 1.45)	0.29	0.86 (0.45, 1.62)	0.63
Ethnicity = Ethnic minorities	1.13 (0.40, 3.22)	0.82	1.12 (0.39, 3.20)	0.83	1.41 (0.58, 3.40)	0.45	1.39 (0.61, 3.20)	0.43	1.36 (0.72, 2.56)	0.35
Smokers (current or ex-smokers)	1.01 (0.34, 3.04)	0.99	3.88 (1.20, 12.5)	**0.023**	1.41 (0.58, 3.46)	0.45	2.19 (0.93, 5.14)	0.07	1.79 (0.92, 3.46)	0.09
BMI (kg/m^2^)	1.08 (0.98, 1.19)	0.10	1.05 (0.95, 1.17)	0.34	1.03 (0.96, 1.11)	0.45	0.98 (0.91, 1.06)	0.59	1.00 (0.94, 1.06)	0.98
CFS on admission	0.80 (0.37, 1.75)	0.58	0.72 (0.34, 1.53)	0.39	1.50 (0.89, 2.53)	0.13	1.14 (0.70, 1.85)	0.59	0.98 (0.66, 1.45)	0.91
**Co-morbidities**										
Cardiac disease (any)	0.30 (0.02, 5.27)	0.41	4.30 (1.13, 16.3)	**0.032**	1.50 (0.35, 6.48)	0.59	1.42 (0.37, 5.43)	0.61	1.60 (0.56, 4.51)	0.38
Chronic kidney disease	-	-	0.95 (0.08, 11.2)	0.97	0.18 (0.01, 2.83)	0.22	1.65 (0.19, 14.35)	0.65	1.23 (0.23, 6.72)	0.81
Diabetes	0.54 (0.09, 3.47)	0.52	0.38 (0.08, 1.84)	0.23	0.37 (0.08, 1.83)	0.22	1.19 (0.29, 4.84)	0.81	0.41 (0.13, 1.25)	0.12
Hypertension	0.20 (0.03, 1.49)	0.12	0.31 (0.07, 1.40)	0.13	1.04 (0.29, 3.70)	0.95	1.14 (0.32, 4.01)	0.84	0.85 (0.32, 2.29)	0.75
Lung condition (any)	0.42 (0.07, 2.64)	0.36	0.47 (0.10, 2.26)	0.35	0.69 (0.18, 2.60)	0.59	1.29 (0.37, 4.50)	0.69	0.90 (0.34, 2.44)	0.84
Mental health condition (any)	-	-	0.40 (0.06, 2.79)	0.36	0.80 (0.17, 3.69)	0.77	4.08 (1.07, 15.6)	**0.040**	1.41 (0.39, 5.07)	0.60
Number of comorbidities	2.64 (0.65, 10.7)	0.18	1.93 (0.91, 4.09)	0.09	1.38 (0.63, 3.03)	0.42	1.02 (0.49, 2.13)	0.97	1.26 (0.70, 2.29)	0.45
**Admission data**										
Number of acute COVID-19 symptoms	0.95 (0.71, 1.27)	0.73	0.99 (0.74, 1.31)	0.93	0.67 (0.51, 0.87)	**0.003**	1.05 (0.85, 1.31)	0.66	0.91 (0.76, 1.08)	0.28
Time from onset of symptoms to time of follow-up appointment (days)	1.01 (1.00, 1.03)	0.16	1.01 (0.99, 1.02)	0.51	0.99 (0.98, 1.01)	0.30	0.99 (0.98, 1.01)	0.46	1.00 (0.99, 1.01)	0.75

BMI: Body mass index; CFS: Clinical frailty scale (using the Rockwood Frailty Scale). Cerebrovascular disease, and immunosuppression were excluded as predictors. *Table data are presented as adjusted odds ratio (aOR) and its 95% confidence ratio (95%CI)

Model selection was made according to clinical and epidemiological reasoning and the Akaike Information Criterion (AIC). This was done separately for each outcome of interest (either one of four specialty referrals and individual specialty referral). Variables were included that improved model fit based on AIC, with a difference of 2 points considered relevant. Total number of Long-COVID symptoms was used rather than the presence of one symptom as this was clinically thought to correlate better with SCR. Regression coefficients are presented as adjusted odds ratios (aOR) for logistic regressions with corresponding 95% confidence interval (95%CI) and two-sided Wald-type statistical test. All tests of significance were two-tailed and a p-value of < 0.05 was considered statistically significant.

## Results

### Characteristics of study subjects

Figure 1 summarises those excluded from follow-up while Supplementary Fig. 1 further breaks down the participant recruitment according to peak admission waves.

### Descriptive data: baseline characteristics and demographics

Post-ED adults were younger (51 (41–63) vs. 61 (51–74), *p* < 0.001) with lower clinical frailty scores (CFS) (2 (2–3) vs. 3 (2–4), *p* < 0.001) compared to PH adults. A greater proportion of post-ED adults were female (47.1% vs. 39.6%, *p* = 0.009) and ethnic minorities (61.6% vs. 48.8%, *p* < 0.001). The prevalence of pre-existing lung conditions was similar between both cohorts but all other pre-existing co-morbidities were lower in post-ED adults.

Post-ED adults had significantly fewer COVID-19 symptoms at hospital attendance (2 (1–4) vs. 3 (2–4), *p* < 0.001) and a lower admission National Early Warning Score 2 (NEWS2) (2 (1–4) vs. 4 (2–6), *p* < 0.001). A lower prevalence of pulmonary embolus (PE) (1.1 vs. 6.1%, *p* < 0.001) was seen in those patients screened for PE in the post-ED group.

### Outcome data: clinical outcomes at follow-up consultation

We achieved follow-up in 687/800 (86%) of PH adults vs. 352/484 (73%) of post-ED adults. PH adults had an earlier clinical review compared to post-ED adults (61 (52–83) vs. 75 days (55–106), *p* < 0.001) and this corresponded with an earlier assessment from the onset of their symptoms (69 (59–92) vs. 85 days (66–112), *p* < 0.001). This assessment falls between 10 and 12 weeks following symptom onset and is in concordance with the group labelled as ‘ongoing symptomatic COVID-19’, as defined by NICE [1]. Table 2 summarises the physical symptoms, mental health outcomes and SCR at follow-up. Supplementary Table 2 summarises the same variables according to Wave 1 and 2 participants.

A smaller proportion of post-ED adults had at least one Long-COVID symptom at follow-up (70.1 vs. 79.5%, *p* < 0.001) but similar total number of Long-COVID symptoms (2 (0–4) vs. 2 (1–4), *p* < 0.001) was observed. Similar prevalence of depressive symptoms was demonstrated (19.1 vs. 19.9%, *p* = 0.74) in post-ED and PH adults respectively. More post-ED adults reported feeling back to normal (74.7 vs. 60.9%, *p* = 0.001) and a greater percentage had returned to work (46.1 vs. 29.4%, *p* < 0.001) at follow up. A greater proportion of chest radiographs in post-ED adults had returned to normal (79.4 vs. 67.9%, *p* < 0.001).

However, post-ED adults were more likely to require any SCR (32.6 vs. 25%, *p* = 0.001) with greater referrals to mental health (15.1 vs. 9.0%, *p* = 0.003) and physiotherapy (14.2% vs. 7.5%, *p* = 0.001) services.

### Subgroup analysis

Across both Waves, and similar to the trend in the overall population, fewer PH adults were back to work or had normal radiology at follow-up. In Wave 1, we observed that post-ED adults required more referrals to mental health services (24.3% vs. 11.2%, *p* = 0.001), while in Wave 2, they were more likely to be referred to physiotherapy (18.0% vs. 9.1%, *p* = 0.002). As the pandemic progressed, PH adults had a shorter time from hospital discharge to follow-up (61 days in Wave 2 vs. 81 days in Wave 1). The opposite occurred in post-ED adults (they were seen at 105 days in Wave 2 vs. 74 days in Wave 1).

### Outcome data: predictors of SCR in the overall cohort

(See Table 3 for adjusted results and Supplementary Table 3 for unadjusted results)

Factors associated with a referral to any one of the four most prevalent specialty referrals (respiratory, cardiology, physiotherapy, or mental health) included a greater total number of Long-COVID symptoms at follow-up (aOR = 1.26, 95%CI: 1.16, 1.36, *p* < 0.001). Unexpectedly, fewer acute COVID-19 symptoms at initial hospital attendance (aOR = 0.87, 95%CI: 0.77, 0.97, *p* = 0.018) were associated with an onward SCR.

Post-ED adults were more likely to be referred to any of the four most prevalent specialty referrals compared to PH adults (aOR = 1.82, 95%CI: 1.19, 2.79, *p* = 0.006) after adjusting for confounders, including the duration between symptom onset to the follow-up appointment, pre-existing mental health and other medical comorbidities. This association was demonstrated for both referrals to physiotherapy (aOR = 2.59, 95%CI: 1.35, 4.96, *p* = 0.004) and mental health services (aOR = 3.84, 95%CI: 2.00, 7.37, *p* < 0.001).

### Outcome data: predictors of SCR in post-ED adults

(See Table 4 for adjusted results and Supplementary Table 4 for unadjusted results)

After adjusting for confounding factors, greater cardiology referrals were associated with those who had smoked (aOR = 3.88, 95%CI: 1.20, 12.5, *p* = 0.023), or had cardiac disease (aOR = 4.30, 95%CI: 1.13, 16.3, *p* = 0.032). Those with a mental health condition (aOR = 4.08, 95%CI: 1.07, 15.6, *p* = 0.040) were more likely to be referred to mental health services. Participants of male gender and those with more acute COVID-19 symptoms at initial presentation required fewer referrals to physiotherapy services at follow-up ((males; aOR = 0.36, 95%CI: 0.15, 0.85, *p* = 0.021), (greater acute symptoms; aOR = 0.67, 95%CI: 0.51, 0.87, *p* = 0.003)).

### Missing data

The study contained missing data in both predictor and outcome variables with BMI (18.3%), depression (14.5%) and post-traumatic stress symptoms (12.7%) containing the most missing values. Listwise deletion was used to remove missing values. Listwise deletion is an approach for dealing with missing data that excludes any participants with at least one missing value from the data analysis. Even though we found associations between missingness on some variables (BMI and number of long-COVID symptoms) and observed information, the statistical analysis results were robust to missingness. Robustness was determined by sensitivity analysis comparing the result from listwise deletion to multiple imputation (a method for dealing with missing values by estimating the unknown values using predictions from a regression model).

## Discussion

### Key findings

This is a prospective UK cohort study that identifies predictors of specialty care referrals (SCR) in post-ED adults with ongoing symptomatic COVID-19. Firstly, we demonstrate post-ED adults have a lower Long-COVID burden than a comparator population discharged from hospital at a median follow-up of 10 to 12 weeks. They are, in addition, more likely to be referred to specialty care services, particularly to psychological services despite similar pre-existing mental health status, and to physiotherapy services despite similar pre-existing lung comorbidity. Secondly, we demonstrate that recognised predictors of Long-COVID previously published by our group (such as pre-existing lung disease or acute COVID-19 burden) [9] do not necessarily predict onward SCR, a more direct marker for healthcare burden. Thirdly, the presence of unique predictors of SCR in post-ED individuals suggests that they are a unique post-COVID cohort with different causal relationships linked to Long-COVID itself and the need for SCR.

A plausible explanation for greater SCR in post-ED adults is that they received less directed recovery during their ED assessment, unlike admitted patients who received holistic inpatient care, particularly from physiotherapy services. This may point to these therapy services being key to addressing Long-COVID recovery. These findings therefore represent real-world post-COVID clinical services, but not all post-ED adults are offered clinical follow-up at hospital trusts across the UK. By using this information, emergency departments can better plan pathways of care for those in need of targeted support. This data can help reshape inequitable access to post-acute COVID-19 care and general post-acute care during future pandemics.

### Evidence in context

Our findings report similar symptom prevalence (70.1%) in a large group of post-ED adults, compared to another UK study by Mallia et al. [17] (70.8%; *n* = 34/48). In comparison to other studies, we examined healthcare resource need in a larger post-ED cohort (*n* = 347) and looked at predictors of specialty care referral, rather than impaired functional recovery. Despite a similar Long-COVID symptom burden in our study (2 (0–4)) compared to Heightman’s study [3] (2 (1–4)), we observed greater SCR in our post-ED group (32.6% vs. 18.9%) though fewer referrals to chronic fatigue services, memory clinics and neurology. This may be due to our exclusion of those too frail to participate in follow-up whereas Heightman et al. included nursing home residents in their cohort. In addition, chronic fatigue services were accessed through community pathways in our service; hence its prevalence may have been understated.

Another study, by Bell et al. [18], evaluated post-COVID adults attending the ED with oxygen saturations > 94% but an exercise desaturation of < 2%. They reported a higher number of onward respiratory referrals of 22.9% (*n* = 44/192) compared to 8.9% in this cohort. This may be due to their earlier follow-up (28 days), a smaller sample size and differences in follow-up protocols. Furthermore, Bell’s cohort presented later in the course of their acute illness (at 13 days of symptoms (IQR 3–28), compared to 7 days (IQR 4–10) here), which equally may have led to greater symptom burden at follow-up.

### Implications for healthcare planning

Our study found unique associations between female sex, pre-existing mental health and cardiac comorbidities, smoking history, and specific SCRs in post-ED individuals following adjustment for confounding variables including co-morbidities. These associations are contrary to predictors of Long-COVID in our cohort of post-hospitalised individuals [9]. This raises a paradox in that predictors of Long-COVID and SCR patterns are independent and variable across different groups of patients. Further understanding in SCR patterns following acute COVID-19 across these unique groups will enable improved clinical pathways, in advance of future pandemics.

Further paradox arises in post-ED individuals as acute symptom burden and Long-COVID symptoms correlate differently with physiotherapy referrals. This highlights that more research into the understanding of the causal relationship between acute disease, ongoing symptomatology and SCR is warranted.

A review article [19] lists predisposing factors in non-hospitalised patients to include female gender, an age between 35 and 69 years, and two or more comorbidities. In the PHOSP-COVID hospitalised cohort [20], female gender, pre-existing mental health and cardiovascular disease were poor predictors of recovery from post-COVID-19 dyspnoea, at five to twelve months. Predictors in our post-ED cohort overlap with both of these groups, suggesting they are an intermediary group worth considering when planning services for future pandemics. We suggest post-ED individuals are offered prompt evaluation of Long-COVID symptoms to accurately determine SCR rates, particularly surrounding mental health (which was less needed in the second wave perhaps due to better coping strategies during the pandemic).

### Strengths of this study

This study compares healthcare resource need, between post-ED and PH adults, which have seldom been studied by past research. Our study has a large sample size in both cohorts, and equally assesses trends in SCR across two timepoints. We offered a comprehensive clinical service that allowed multidisciplinary health care professionals (nursing staff, doctors, physiotherapists, and occupational therapists) to best support individuals with complex health needs via a standardised approach, ensuring equitable healthcare for all reached by the service. This model of care should be used to support recovery in future pandemics.

We attempted to minimise selection and follow-up bias through offering all eligible patients follow-up according to standardised protocols. Our analysis also accounted for differences in symptom profile that may have arisen from variation in the time between the onset of COVID-19 symptoms and our assessment, thereby minimising the possibility of an inflated symptom burden due to a longer follow-up interval from discharge if, for example, anxiety or post-traumatic stress symptoms were not managed in the interim.

### Limitations of this study

Limitations include single centre data and unequal cohort sizes. Selection bias exists within the cohort as only patients with abnormal radiological appearances were included within this study. Equally, a high proportion of patients were unable to be reached at follow-up, with post-ED adults harder to reach (27% lost to follow up) as compared to PH adults (14% lost to follow up). This may be attributable to contact numbers being more reliably sourced for hospitalised inpatients. PH adults equally had a greater expectation of follow-up prior to discharge than the post-ED group which may have resulted in greater engagement with phone calls following discharge. This may therefore lead to underrepresented outcomes in the post-ED group regarding SCR referral rates (if patients could not seek support) or inflated outcomes (if participants were well and therefore did not need to engage with healthcare services). However, this study contributes important data despite acknowledgement of these limitations.

The decision to refer to a particular specialty was also subject to bias, as although standardised protocols were used, clinicians were aware of whether patients had been seen in the ED only or admitted to hospital. SCR may have therefore been underestimated, particularly to mental health services, as the TSQ questionnaire [21] may have limitations in holistically evaluating anxiety from other causes beyond the acute reactive stress of the COVID-19 pandemic. Equally, greater referrals to specific specialties may have occurred in participants with the same baseline pre-existing co-morbidity; however, our analysis adjusted for these confounding factors. Additionally, we recognise that although baseline clinical frailty was included as a possible confounder, there are limitations in using the Rockwood Clinical Frailty score. This tool is validated for use in older persons over 65 [22] with less applicability across a younger cohort [23] such as that observed in this study (median age 51 to 61 years).

Lastly, ward patients with more severe disease and prolonged ICU stays may have remained in hospital at data capture, limiting their outcome representation. Post-COVID clinics are now established nationwide and offered to both non-hospitalised and hospitalised individuals. Collaborative data from these services is needed to validate if similar predictors of referrals are seen in post-ED adults as compared to our study.

## Conclusion

Our research presents real world data from the early-to-mid stages of the COVID-19 pandemic in an under-represented cohort and poorly evaluated research population with unique healthcare needs. In summary, we identify unique predictors of ongoing symptomatic COVID-19 in a group of post-ED adults who, despite experiencing moderate acute disease, often do not receive further clinical follow-up. The identification of a considerable need for onward referral to specialty services amongst post-ED adults argues for their recognition as a distinct group within Long-COVID support service planning who deserve specific and targeted support. It is imperative that this gap in preparedness is addressed before future pandemics arise.

## Electronic supplementary material

Below is the link to the electronic supplementary material.

Supplementary Material 1

## Data Availability

All data relevant to the study are included in this published article or uploaded as supplementary information.

## Abbreviations

95%CI: 95% confidence interval
AIC: Akaike Information Criterion
aOR: Adjusted odds ratio
ARC: ‘Assessing Recovery from COVID-19’ consortium (North Central London)
BMI: Body mass index
BSTI: British Society of Thoracic Imaging
CFS: Rockwood Clinical Frailty Score
COVID-19: Coronavirus disease 2019
ICU: Intensive care unit
IQR: Interquartile range
NEWS2: National Early Warning Score 2
NHS: National Health Service (United Kingdom)
NICE: National Institute for Health and Care Excellence (United Kingdom)
PE: Pulmonary embolus
PH: Post-hospitalised
PHOSP-COVID: Post-hospitalisation COVID-19 study
PHQ-2: Patient Health Questionnaire-2
Post-ED: Post-Emergency Department
SARS-CoV-2: Severe acute respiratory syndrome coronavirus 2
SCR: Specialist care referrals
STROBE: Strengthening the Reporting of Observational Studies in Epidemiology
TSQ: Trauma Screening Questionnaire
VAS: Visual analogue scale
