# Supplementary material for: Predictors of specialist care referrals (SCR) following emergency department review or hospital admission in adults with previous acute COVID-19: a prospective UK cohort study
Source: BMC Emerg Med. 2025 Jan 23;25:11. doi: 10.1186/s12873-024-01164-x (PMC11756147; doi:10.1186/s12873-024-01164-x)
Supplement: Supplementary file 1 — Supplementary Material 1 [file 12873_2024_1164_MOESM1_ESM.docx]

**Supplementary Material**

**Supplementary Figure 1: Study participants in PH and post-ED groups across Wave 1 and 2**

**
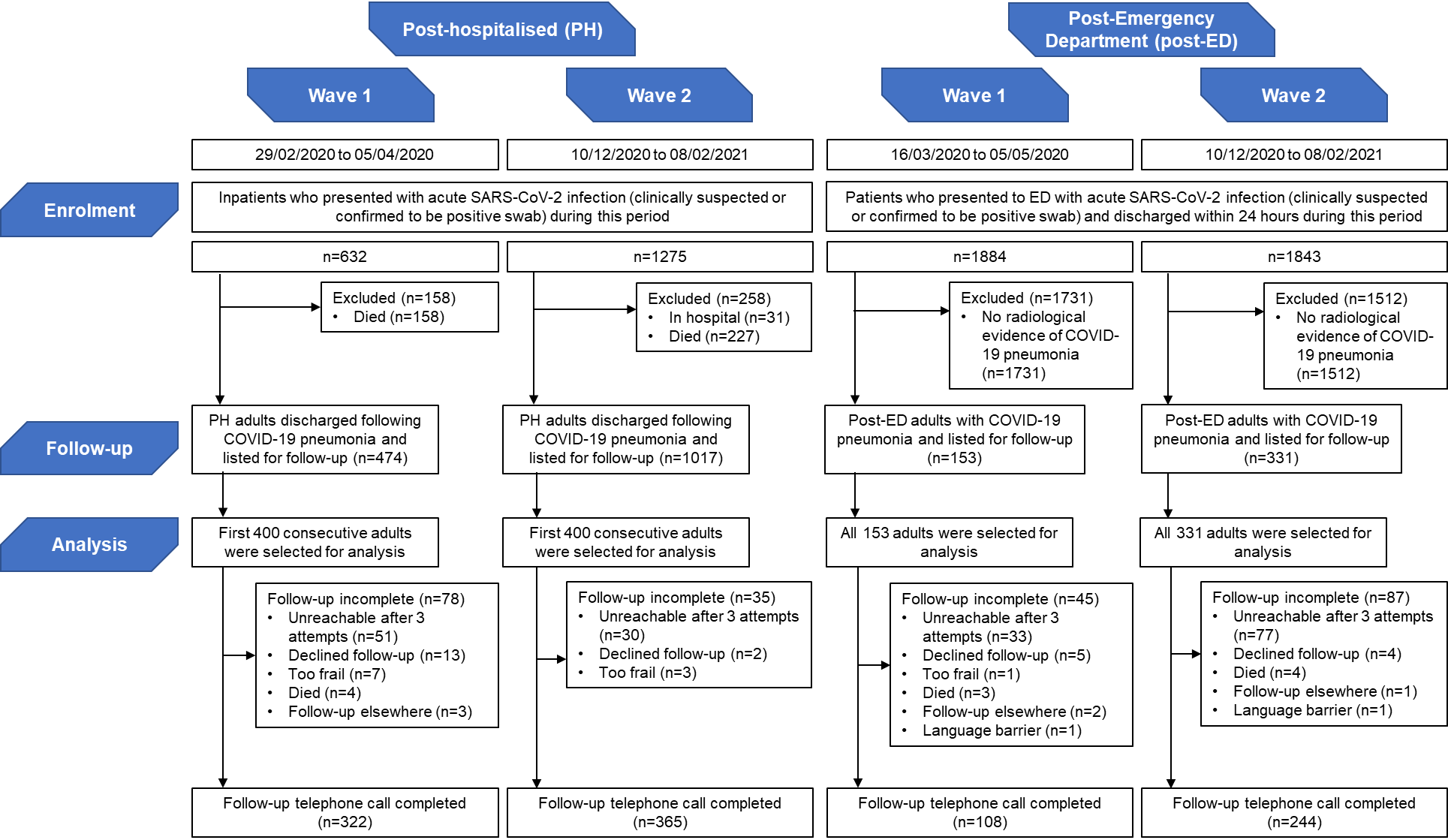
**

**Supplementary Figure 2: Protocolised pathway for managing persistent respiratory symptoms in Long-COVID patients**


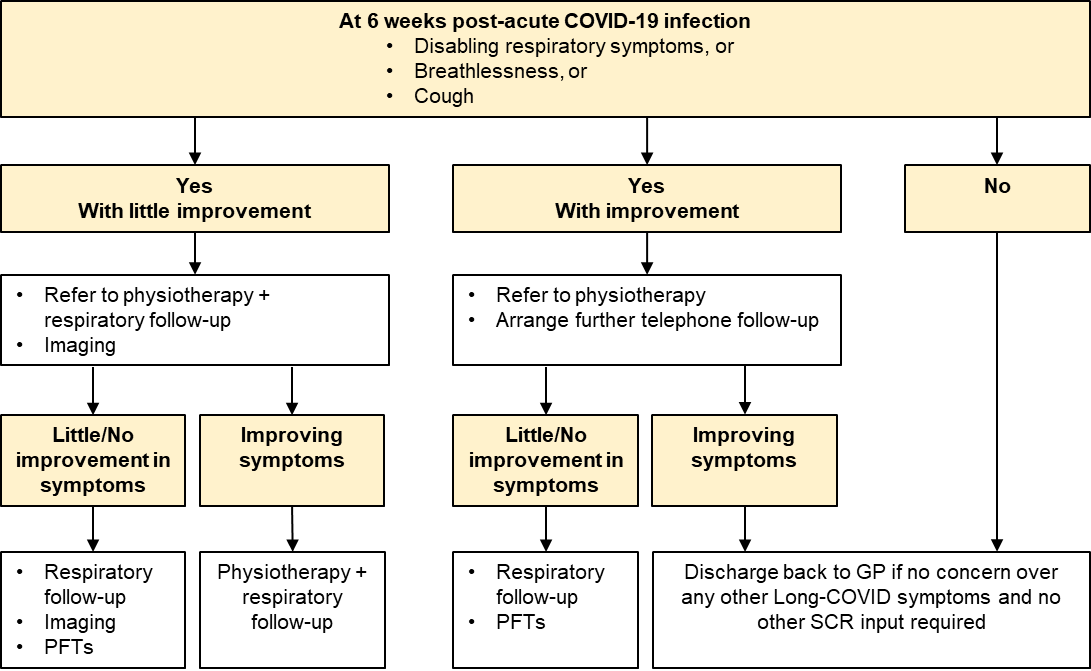


PFTs: Pulmonary function tests

**Supplementary Figure 3: Protocolised pathway for managing disabling chest pain in Long-COVID patients**

**
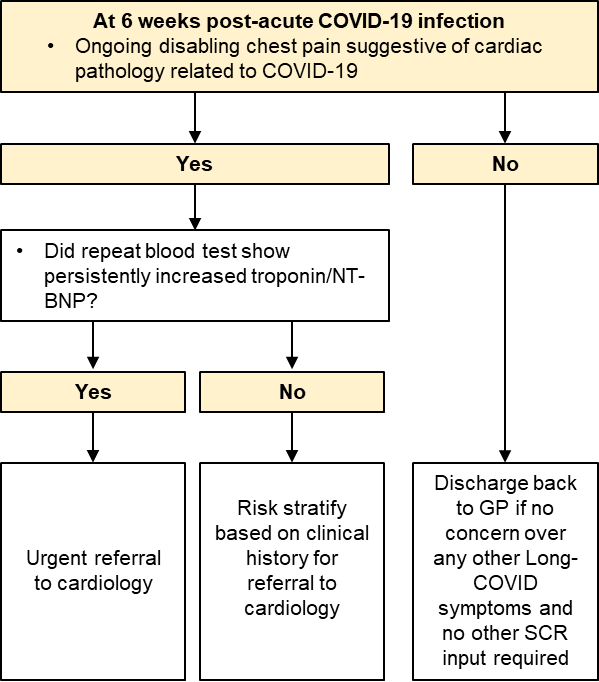
**

**Supplementary Figure 4: Protocolised pathway for managing psychological symptoms in Long-COVID patients**

**
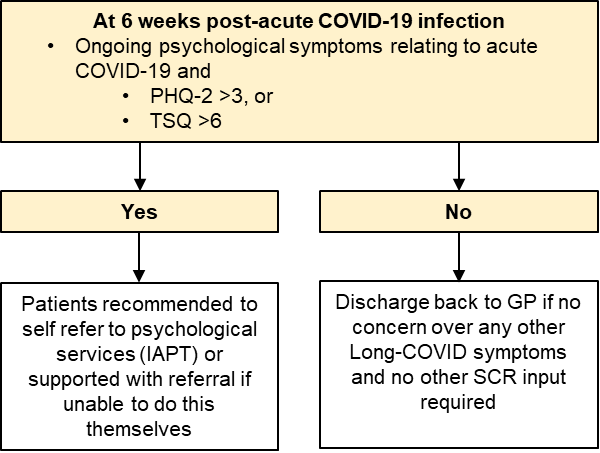
**

**Supplementary Table 1: Blood parameters at admission comparing PH and post-ED patients**

|  | **At admission** | | | **At follow-up** | | |
| --- | --- | --- | --- | --- | --- | --- |
| **Blood parameter** | **PH**  **N=800** | **Post-ED**  **N=484** | **p-value** | **PH**  **N=800** | **Post-ED**  **N=484** | **p-value** |
| White cells (×10^9^/L) | N=691  6.57 (5.00-8.82) | N=399  5.76 (4.59-7.60) | **<0.001** | N=411  6.86 (5.78-8.32) | N=274  6.78 (5.27-8.30) | 0.14 |
| Platelets (×10^9^/L) | N=710  210 (168-267) | N=399  209 (166-269) | 0.87 | N=412  260 (216-306) | N=274  256 (209-310) | 0.50 |
| Neutrophils (×10^9^/L) | N=691  5.03 (3.51-7.07) | N=399  3.97 (2.90-5.34) | **<0.001** | N=411  3.83 (2.89-4.84) | N=274  3.74 (2.73-4.99) | 0.37 |
| Lymphocytes (×10^9^/L) | N=691  0.97 (0.68-1.32) | N=399  1.17 (0.89-1.58) | **<0.001** | N=412  2.08 (1.55-2.64) | N=274  2.07 (1.55-2.55) | 0.49 |
| Eosinophils (×10^9^/L) | N=712  0 (0-0.02) | N=399  0.01 (0-0.04) | **<0.001** | N=411  0.15 (0.10-0.26) | N=274  0.13 (0.08-0.22) | **0.003** |
| D-dimer (ng/mL) | N=530  855 (544-1500) | N=315  587 (391-947) | **<0.001** | N=371  430 (264-795) | N=210  386 (279-639) | 0.16 |
| Ferritin (μg/L) | N=551  761 (423-1470) | N=346  421 (178.5-898) | **<0.001** | N=373  157 (77-311) | N=242  121 (50-287) | **0.012** |
| Urea (mmol/L) | N=703  5.6 (4.0-8.3) | N=364  4.4 (3.2-5.8) | **<0.001** | N=303  5.4 (4.4-7.0) | N=197  4.9 (3.8-6.4) | **<0.001** |
| Creatinine (μmol/L) | N=680  85 (69-107) | N=364  76 (64-93) | **<0.001** | N=303  77 (66-93) | N=197  74 (62-88) | **0.028** |
| Bilirubin (μmol/L) | N=680  8 (6-11) | N=367  7 (5-10) | **<0.001** | N=408  7 (5-10) | N=257  6 (4-9) | **0.035** |
| ALT (IU/L) | N=613  33 (22-58) | N=353  33 (24-53) | 0.81 | N=407  24 (18-36) | N=255  25 (17-38) | 0.68 |
| AST (IU/L) | N=418  45 (32-68) | N=219  38 (26-50) | **<0.001** | N=386  23 (19-29) | N=226  24 (20-30) | 0.25 |
| Glucose (mmol/L) | N=550  6.4 (5.6-8.0) | N=314  6.1 (5.4-7.0) | **<0.001** | N=370  5.8 (5.0-7.3) | N=228  5.5 (4.9-6.5) | **0.004** |
| C-reactive protein (mg/dL) | N=686  75 (34-137) | N=386  29 (12-70) | **<0.001** | N=408  2 (1-5) | N=248  2 (1-6) | **0.004** |
| Creatine kinase (IU/L) | N=235  163 (91-325) | N=321  103 (61-182) | **<0.001** | N=182  88 (59-132) | N=240  100 (68-155) | **0.007** |
| Troponin T (ng/L) | N=523  11 (6-24) | N=301  6 (4-11) | **<0.001** | N=369  8 (5-15) | N=214  6 (4-10) | **0.003** |
| BNP (ng/L) | N=361  177 (54-528) | N=155  115 (69-276) | 0.28 | N=370  72 (50-195) | N=117  105 (65-253) | **<0.001** |

**Table data are presented as median (IQR) for continuous data that is not normally distributed, and as number (N) and percentage (%) for categorical data. Two-tailed Student’s t-test was used for normally distributed continuous data. Mann-Whitney-U test was used for non-normally distributed continuous data. Chi-squared test was used for categorical data.*

**Supplementary Table 2: Clinical Outcomes at initial follow-up consultation for post-ED and PH adults according to time of admission**

|  | **Wave 1**  Admission date: 29/02/2020 – 05/04/2020 | | | **Wave 2**  Admission date: 10/12/2020 – 08/02/2021 | | |
| --- | --- | --- | --- | --- | --- | --- |
| **Variable** | **PH** | **Post-ED** | **p-value** | **PH** | **Post-ED** | **p-value** |
| Time from hospital discharge/ED attendance to follow-up appointment (days) | N=309  81 (67-104) | N=102  74 (67-85) | **<0.001** | N=295  61 (53-73) | N=202  105 (64-119) | **<0.001** |
| Time from onset of symptoms to time of follow-up appointment (days) | N=323  74 (60-97) | N=106  64 (57-76) | **<0.001** | N=354  54 (46-66) | N=241  99 (53-110) | **<0.001** |
| **Symptom burden at follow-up** | | | | | | |
| Any 1 of 14 Long-COVID symptoms at follow-up | 263/317 (83.0%) | 78/104 (75.0%) | 0.07 | 271/355 (76.3%) | 164/241 (68.0%) | **0.025** |
| Total number of symptoms at follow-up | N=317  3 (1-5) | N=105  2 (0-4) | 0.10 | N=358  2 (1-4) | N=241  2 (0-3) | **0.025** |
| **Mental Health Burden** | | | | | | |
| Depressive symptoms highlighted by Patient Health Questionnaire-2 Score ≥ 2 | 87/382 (22.8%) | 27/151 (17.9%) | 0.21 | 42/265 (15.8%) | 38/237 (16.0%) | 0.96 |
| Post-traumatic stress symptoms highlight by Trauma Screening Questionnaire Score ≥ 6 | 27/346 (7.8%) | 11/104 (10.6%) | 0.37 | 11/258 (4.3%) | 1/241 (0.4%) | **0.005** |
| **Functional Recovery** | | | | | | |
| Patients reporting feeling back to normal | 160/313 (51.1%) | 65/106 (61.3%) | 0.07 | 111/132 (84.1%) | 98/113 (86.7%) | 0.56 |
| Number of patients back to work  Yes  No  N/A | 76/308 (24.7%)  73/308 (23.7%)  159/308 (51.6%) | 43/102 (42.2%)  21/102 (20.6%)  38/102 (37.3%) | **0.003** | 114/339 (33.6%)  81/339 (23.9%)  144/339 (42.5%) | 112/234 (47.9%)  48/234 (20.5%)  74/234 (31.6%) | **0.002** |
| **Radiology** | | | | | | |
| Chest X-ray result  Normal  Significantly improved (PCVCX1) | 212/309 (68.6%)  55/309 (17.8%) | 45/54 (83.3%)  6/54 (11.1%) | **0.017** | 187/279 (67.0%)  65/279 (23.3%) | 156/199 (78.4%)  34/199 (17.1%) | **0.017** |
| **Specialty care referral (SCR)** | | | | | | |
| Any 1 of 4 referrals (Respiratory/cardiology/physiotherapy/mental health) | 70/286 (24.5%) | 32/106 (30.2%) | 0.25 | 86/339 (25.4%) | 82/244 (33.6%) | **0.030** |
| Respiratory referrals | 27/280 (9.6%) | 5/106 (4.7%) | 0.12 | 36/339 (10.6%) | 26/244 (10.7%) | 0.99 |
| Cardiology referrals | 26/287 (9.1%) | 9/107 (8.4%) | 0.84 | 42/339 (12.4%) | 23/244 (9.4%) | 0.26 |
| Physiotherapy referrals for exercise rehabilitation and/or management of dysfunctional breathing | 16/287 (5.6%) | 6/107 (5.6%) | 0.99 | 31/339 (9.1%) | 44/244 (18.0%) | **0.002** |
| Mental health referrals | 32/286 (11.2%) | 26/107 (24.3%) | **0.001** | 24/339 (7.1%) | 27/244 (11.1%) | 0.09 |
| Neurology referrals | 1/287 (0.3%) | 0/107 (0.0%) | 0.55 | 0/339 (0.0%) | 0/244 (0.0%) | - |
| Chronic fatigue service referrals | 2/287 (0.7%) | 0/107 (0.0%) | 0.40 | 0/339 (0.0%) | 0/244 (0.0%) | - |
| Memory clinic referrals | 2/287 (0.7%) | 0/107 (0.0%) | 0.40 | 1/339 (0.3%) | 0/244 (0.0%) | 0.40 |

**Table data are presented as median (IQR) for continuous data that is not normally distributed, and as number (N) and percentage (%) for categorical data. Two-tailed Student’s t-test was used for normally distributed continuous data. Mann-Whitney-U test was used for non-normally distributed continuous data. Chi-squared test was used for categorical data.*

**Supplementary Table 3: Multiple Logistic Regression Model for specialty care referrals (SCR) post-discharge in all patients**

| **Referral Outcome** | **Respiratory (N=614)** | | | | **Cardiology (N=619)** | | | | **Physiotherapy (N=619)** | | | | **Mental health (N=618)** | | | | **At least one of four referrals (N=614)** | | | |
| --- | --- | --- | --- | --- | --- | --- | --- | --- | --- | --- | --- | --- | --- | --- | --- | --- | --- | --- | --- | --- |
| **Predictor Variable** | **Adjusted** | | **Unadjusted** | | **Adjusted** | | **Unadjusted** | | **Adjusted** | | **Unadjusted** | | **Adjusted** | | **Unadjusted** | | **Adjusted** | | **Unadjusted** | |
|  | **OR**  **(95 % CI)** | **p-value** | **OR**  **(95 % CI)** | **p-value** | **OR**  **(95 % CI)** | **p-value** | **OR**  **(95 % CI)** | **p-value** | **OR**  **(95 % CI)** | **p-value** | **OR**  **(95 % CI)** | **p-value** | **OR**  **(95 % CI)** | **p-value** | **OR**  **(95 % CI)** | **p-value** | **OR**  **(95 % CI)** | **p-value** | **OR**  **(95 % CI)** | **p-value** |
| **Long-COVID symptoms** | | | | | | | | | | | | | | | | | | | | |
| Number of Long-COVID symptoms (at follow-up) | 1.27  (1.13, 1.41) | **<0.001** | 1.23  (1.11, 1.36) | **<0.001** | 1.13  (1.00, 1.27) | 0.05 | 1.12  (1.02, 1.22) | **0.012** | 1.23  (1.09, 1.38) | **<0.001** | 1.20  (1.11, 1.31) | **<0.001** | 1.34  (1.19, 1.50) | **<0.001** | 1.31  (1.21, 1.43) | **<0.001** | 1.26  (1.16, 1.36) | **<0.001** | 1.23  (1.14, 1.32) | **<0.001** |
| **Patient characteristics** | | | | | | | | | | | | | | | | | | | | |
| Post-ED | 0.82  (0.43, 1.58) | 0.56 | 0.78  (0.44, 1.36) | 0.37 | 1.39  (0.73, 2.65) | 0.32 | 0.86  (0.49, 1.51) | 0.60 | 2.59  (1.35, 4.96) | **0.004** | 2.45  (1.43, 4.21) | **0.001** | 3.84  (2.00, 7.37) | **<0.001** | 2.68  (1.57, 4.57) | **<0.001** | 1.82  (1.19, 2.79) | **0.006** | 1.39  (0.98, 1.99) | 0.07 |
| Age (years) | 1.00  (0.98, 1.03) | 0.82 | 1.00  (0.99, 1.02) | 0.80 | 1.03  (1.00, 1.06) | **0.023** | 1.03  (1.01, 1.04) | **0.005** | 1.02  (0.99, 1.04) | 0.22 | 1.00  (0.99, 1.02) | 0.69 | 1.01  (0.98, 1.03) | 0.72 | 1.00  (0.98, 1.02) | 0.85 | 1.02  (1.00, 1.03) | 0.06 | 1.01  (1.00, 1.02) | 0.06 |
| Sex = Male | 1.52  (0.82, 2.82) | 0.18 | 1.41  (0.80, 2.50) | 0.24 | 1.03  (0.56, 1.88) | 0.93 | 1.00  (0.57, 1.76) | 0.99 | 0.81  (0.45, 1.45) | 0.48 | 0.55  (0.33, 0.94) | **0.029** | 0.67  (0.37, 1.21) | 0.18 | 0.62  (0.37, 1.04) | 0.07 | 1.04  (0.70, 1.54) | 0.84 | 0.82  (0.57, 1.17) | 0.28 |
| Ethnicity = Ethnic minorities | 0.72  (0.40, 1.30) | 0.27 | 0.76  (0.44, 1.30) | 0.31 | 0.91  (0.49, 1.66) | 0.74 | 0.79  (0.46, 1.38) | 0.41 | 1.28  (0.70, 2.33) | 0.42 | 1.03  (0.61, 1.76) | 0.90 | 1.09  (0.59, 2.01) | 0.78 | 1.07  (0.64, 1.80) | 0.80 | 0.93  (0.62, 1.37) | 0.70 | 0.88  (0.62, 1.25) | 0.47 |
| Ever smoked | 0.77  (0.43, 1.38) | 0.38 | 0.93  (0.53, 1.62) | 0.79 | 1.27  (0.71, 2.30) | 0.42 | 1.41  (0.81, 2.45) | 0.23 | 0.97  (0.53, 1.75) | 0.91 | 0.95  (0.55, 1.63) | 0.85 | 2.04  (1.11, 3.74) | **0.022** | 1.55  (0.92, 2.60) | 0.10 | 1.09  (0.94, 1.60) | 0.68 | 1.14  (0.79, 1.63) | 0.49 |
| BMI (kg/m^2^) | 1.02  (0.96, 1.07) | 0.59 | 1.00  (0.96, 1.05) | 0.87 | 1.03  (0.98, 1.09) | 0.28 | 1.02  (0.97, 1.06) | 0.47 | 1.07  (1.02, 1.13) | **0.010** | 1.01  (0.97, 1.06) | 0.64 | 1.00  (0.94, 1.05) | 0.85 | 0.97  (0.93, 1.02) | 0.20 | 1.00  (0.96, 1.04) | 0.89 | 0.98  (0.95, 1.01) | 0.13 |
| CFS on admission | 1.05  (0.80, 1.39) | 0.71 | 1.07  (0.87, 1.31) | 0.53 | 0.86  (0.65, 1.15) | 0.32 | 1.08  (0.88, 1.33) | 0.46 | 1.17  (0.88, 1.54) | 0.28 | 1.18  (0.98, 1.43) | 0.08 | 0.85  (0.63, 1.16) | 0.32 | 0.99  (0.81, 1.22) | 0.95 | 0.91  (0.76, 1.09) | 0.31 | 1.08  (0.94, 1.24) | 0.26 |
| **Co-morbidities** | | | | | | | | | | | | | | | | | | | | |
| Cardiac disease (any) | 0.86  (0.26, 2.88) | 0.81 | 1.07  (0.51, 2.26) | 0.86 | 1.52  (0.56, 4.15) | 0.41 | 2.40  (1.26, 4.55) | **0.007** | 0.78  (0.25, 2.41) | 0.66 | 1.03  (0.49, 2.16) | 0.95 | 2.59  (1.00, 6.67) | 0.05 | 1.77  (0.93, 3.37) | 0.08 | 1.19  (0.59, 2.14) | 0.63 | 1.36  (0.84, 2.20) | 0.21 |
| Cerebrovascular disease | 0.30  (0.03, 2.84) | 0.29 | 0.39  (0.05, 2.97) | 0.37 | 1.45  (0.36, 5.73) | 0.60 | 1.97  (0.65, 5.96) | 0.23 | 0.33  (0.06, 1.92) | 0.22 | 0.78  (0.18, 3.40) | 0.74 | 1.65  (0.33, 8.23) | 0.54 | 1.68  (0.56 5.06) | 0.36 | 1.24  (0.44, 3.50) | 0.69 | 1.84  (0.80, 4.23) | 0.15 |
| Chronic kidney disease | 0.57  (0.12, 2.64) | 0.47 | 0.88  (0.30, 2.54) | 0.81 | 0.29  (0.06, 1.35) | 0.11 | 0.66  (0.20, 2.21) | 0.50 | 0.26  (0.06, 1.23) | 0.09 | 0.38  (0.09, 1.62) | 0.19 | 0.76  (0.16, 3.56) | 0.72 | 0.57  (0.17, 1.88) | 0.35 | 0.65  (0.26, 1.64) | 0.36 | 0.77  (0.38, 1.56) | 0.47 |
| Diabetes | 1.08  (0.37, 3.15) | 0.89 | 1.05  (0.54, 2.05) | 0.88 | 0.82  (0.31, 2.18) | 0.69 | 1.13  (0.58, 2.20) | 0.73 | 0.30  (0.10, 0.89) | **0.031** | 0.59  (0.27, 1.27) | 0.18 | 1.01  (0.36, 2.85) | 0.99 | 0.74  (0.37, 1.50) | 0.41 | 0.76  (0.38, 1.52) | 0.44 | 0.81  (0.52, 1.29) | 0.38 |
| Hypertension | 0.79  (0.28, 2.27) | 0.66 | 1.07  (0.61, 1.87) | 0.82 | 0.72  (0.29, 1.83) | 0.49 | 1.47  (0.84, 2.56) | 0.18 | 0.55  (0.22, 1.41) | 0.21 | 1.07  (0.62, 1.86) | 0.81 | 1.29  (0.51, 3.28) | 0.59 | 1.07  (0.62, 1.84) | 0.80 | 0.86  (0.46, 1.64) | 0.65 | 1.18  (0.82, 1.71) | 0.37 |
| Immunosuppressed | 0.74  (0.17, 3.28) | 0.69 | 0.70  (0.21, 2.34) | 0.56 | 1.25  (0.37, 4.17) | 0.72 | 1.74  (0.70, 4.34) | 0.23 | 0.59  (0.14, 2.42) | 0.46 | 0.68  (0.21, 2.28) | 0.54 | 0.55  (0.13, 2.29) | 0.41 | 0.64  (0.19, 2.15) | 0.47 | 0.86  (0.36, 2.03) | 0.72 | 0.88  (0.43, 1.80) | 0.73 |
| Lung condition (any) | 0.58  (0.17, 1.91) | 0.37 | 0.83  (0.41, 1.69) | 0.61 | 0.59  (0.20, 1.75) | 0.34 | 1.00  (0.50, 1.99) | 1.00 | 0.66  (0.24, 1.81) | 0.42 | 1.38  (0.74, 2.56) | 0.31 | 0.82  (0.28, 2.43) | 0.73 | 0.83  (0.42, 1.64) | 0.59 | 0.70  (0.34, 1.45) | 0.34 | 0.97  (0.62, 1.51) | 0.89 |
| Mental health condition (any) | 0.33  (0.08, 1.41) | 0.14 | 0.71  (0.25, 2.04) | 0.52 | 0.43  (0.12, 1.54) | 0.20 | 0.73  (0.26, 2.11) | 0.57 | 0.65  (0.21, 2.02) | 0.46 | 1.08  (0.44, 2.62) | 0.87 | 2.49  (0.91, 6.81) | 0.08 | 2.65  (1.32, 5.34) | **0.006** | 1.05  (0.49, 2.24) | 0.91 | 1.49  (0.83, 2.66) | 0.18 |
| Number of comorbidities | 1.18  (0.54, 2.57) | 0.69 | 0.98  (0.80, 1.21) | 0.87 | 1.30  (0.70, 2.42) | 0.41 | 1.20  (0.99, 1.44) | 0.06 | 1.65  (0.88, 3.08) | 0.12 | 0.99  (0.81, 1.21) | 0.94 | 0.89  (0.48, 1.62) | 0.69 | 0.99  (0.82, 1.21) | 0.93 | 1.15  (0.73, 1.80) | 0.55 | 1.05  (0.92, 1.19) | 0.50 |
| **Admission data** | | | | | | | | | | | | | | | | | | | | |
| Number of acute COVID-19 symptoms | 0.88  (0.74, 1.04) | 0.14 | 0.93  (0.80, 1.09) | 0.39 | 0.94  (0.79, 1.12) | 0.47 | 0.94  (0.80, 1.11) | 0.94 | 0.75  (0.62, 0.90) | **0.002** | 0.74  (0.62, 0.88) | **0.001** | 0.94  (0.80, 1.12) | 0.51 | 1.00  (0.86, 1.16) | 0.98 | 0.87  (0.77, 0.97) | **0.018** | 0.91  (0.82, 1.01) | 0.07 |
| Time from onset of symptoms to time of follow-up appointment (days) | 1.00  (0.99, 1.01) | 0.68 | 1.00  (0.99, 1.01) | 0.81 | 0.99  (0.98, 1.00) | 0.06 | 0.99  (0.98, 1.00) | 0.09 | 1.00  (0.99, 1.01) | 0.47 | 1.00  (0.99, 1.01) | 0.90 | 0.99  (0.98, 1.00) | 0.10 | 0.99  (0.98, 1.01) | 0.29 | 0.99  (0.99, 1.00) | 0.06 | 1.00  (0.99, 1.00) | 0.17 |

*BMI: Body mass index; CFS: Clinical frailty scale (measured by Rockwood Frailty Scale). *Table data are presented as adjusted odds ratio (aOR) and its 95% confidence ratio (95%CI).*

**Supplementary Table 4: Multiple Logistic Regression Model for specialty care referrals (SCR) post-discharge in post-ED cohort**

| **Referral Outcome** | **Respiratory (N=245)** | | | | **Cardiology (N=245)** | | | | **Physiotherapy (N=245)** | | | | **Mental health (N=245)** | | | | **At least one of four referrals (N=245)** | | | |
| --- | --- | --- | --- | --- | --- | --- | --- | --- | --- | --- | --- | --- | --- | --- | --- | --- | --- | --- | --- | --- |
| **Predictor Variable** | **Adjusted** | | **Unadjusted** | | **Adjusted** | | **Unadjusted** | | **Adjusted** | | **Unadjusted** | | **Adjusted** | | **Unadjusted** | | **Adjusted** | | **Unadjusted** | |
|  | **OR**  **(95 % CI)** | **p-value** | **OR**  **(95 % CI)** | **p-value** | **OR**  **(95 % CI)** | **p-value** | **OR**  **(95 % CI)** | **p-value** | **OR**  **(95 % CI)** | **p-value** | **OR**  **(95 % CI)** | **p-value** | **OR**  **(95 % CI)** | **p-value** | **OR**  **(95 % CI)** | **p-value** | **OR**  **(95 % CI)** | **p-value** | **OR**  **(95 % CI)** | **p-value** |
| **Long-COVID symptoms** | | | | | | | | | | | | | | | | | | | | |
| Number of Long-COVID symptoms (at follow-up) | 1.43  (1.15, 1.77) | **0.001** | 1.30  (1.11, 1.53) | **0.001** | 1.37  (1.09, 1.70) | **0.006** | 1.18  (1.02, 1.35) | **0.024** | 1.35  (1.12, 1.62) | **0.002** | 1.31  (1.16, 1.48) | **<0.001** | 1.39  (1.17, 1.64) | **<0.001** | 1.42  (1.25, 1.60) | **<0.001** | 1.42  (1.23, 1.65) | **<0.001** | 1.41  (1.24, 1.61) | **<0.001** |
| **Patient characteristics** | | | | | | | | | | | | | | | | | | | | |
| Age (years) | 1.02  (0.97, 1.07) | 0.45 | 1.00  (0.97, 1.03) | 0.99 | 1.05  (1.00, 1.10) | 0.06 | 1.03  (1.00, 1.06) | **0.047** | 1.00  (0.97, 1.04) | 0.91 | 1.01  (0.99, 1.04) | 0.32 | 0.99  (0.96, 1.02) | 0.50 | 1.00  (0.98, 1.03) | 0.72 | 1.01  (0.99, 1.04) | 0.40 | 1.02  (1.00, 1.03) | 0.09 |
| Sex = Male | 1.38  (0.48, 3.98) | 0.55 | 1.11  (0.45, 2.74) | 0.82 | 1.47  (0.49, 4.40) | 0.49 | 1.65  (0.64, 4.24) | 0.30 | 0.36  (0.15, 0.85) | **0.021** | 0.37  (0.18, 0.77) | **0.008** | 0.64  (0.28, 1.45) | 0.29 | 0.67  (0.34, 1.33) | 0.25 | 0.86  (0.45, 1.62) | 0.63 | 0.75  (0.44, 1.28) | 0.30 |
| Ethnicity = Ethnic minorities | 1.13  (0.40, 3.22) | 0.82 | 0.93  (0.38, 2.30) | 0.88 | 1.12  (0.39, 3.20) | 0.83 | 0.75  (0.31, 1.83) | 0.52 | 1.41  (0.58, 3.40) | 0.45 | 0.90  (0.44, 1.82) | 0.77 | 1.39  (0.61, 3.20) | 0.43 | 1.19  (0.59, 2.39) | 0.62 | 1.36  (0.72, 2.56) | 0.35 | 1.02  (0.59, 1.74) | 0.95 |
| Ever smoked | 1.01  (0.34, 3.04) | 0.99 | 0.94  (0.38, 2.36) | 0.90 | 3.88  (1.20, 12.5) | **0.023** | 2.62  (1.05, 6.58) | **0.040** | 1.41  (0.58, 3.46) | 0.45 | 0.78  (0.38, 1.61) | 0.49 | 2.19  (0.93, 5.14) | 0.07 | 1.26  (0.64, 2.49) | 0.50 | 1.79  (0.92, 3.46) | 0.09 | 1.14  (0.67, 1.95) | 0.63 |
| BMI (kg/m^2^) | 1.08  (0.98, 1.19) | 0.10 | 1.03  (0.96, 1.10) | 0.48 | 1.05  (0.95, 1.17) | 0.34 | 1.03  (0.96, 1.10) | 0.47 | 1.03  (0.96, 1.11) | 0.45 | 0.97  (0.93, 1.02) | 0.24 | 0.98  (0.91, 1.06) | 0.59 | 0.97  (0.92, 1.01) | 0.12 | 1.00  (0.94, 1.06) | 0.98 | 0.97  (0.93, 1.01) | 0.10 |
| CFS on admission | 0.80  (0.37, 1.75) | 0.58 | 1.15  (0.73, 1.81) | 0.55 | 0.72  (0.34, 1.53) | 0.39 | 0.95  (0.58, 1.57) | 0.85 | 1.50  (0.89, 2.53) | 0.13 | 1.41  (1.01, 1.98) | **0.046** | 1.14  (0.70, 1.85) | 0.59 | 1.34  (0.96, 1.87) | 0.09 | 0.98  (0.66, 1.45) | 0.91 | 1.30  (0.98, 1.73) | 0.07 |
| **Co-morbidities** | | | | | | | | | | | | | | | | | | | | |
| Cardiac disease (any) | 0.30  (0.02, 5.27) | 0.41 | 0.38  (0.05, 2.96) | 0.36 | 4.30  (1.13, 16.3) | **0.032** | 3.98  (1.40, 11.4) | **0.010** | 1.50  (0.35, 6.48) | 0.59 | 1.01  (0.33, 3.10) | 0.99 | 1.42  (0.37, 5.43) | 0.61 | 1.23  (0.44, 3.46) | 0.70 | 1.60  (0.56, 4.51) | 0.38 | 1.27  (0.55, 2.91) | 0.58 |
| Chronic kidney disease | - | - | - | - | 0.95  (0.08, 11.2) | 0.97 | 1.35  (0.16, 11.4) | 0.78 | 0.18  (0.01, 2.83) | 0.22 | 0.77  (0.09, 6.35) | 0.06 | 1.65  (0.19, 14.35) | 0.65 | 1.59  (0.32, 7.95) | 0.57 | 1.23  (0.23, 6.72) | 0.81 | 0.56  (0.15, 2.15) | 0.40 |
| Diabetes | 0.54  (0.09, 3.47) | 0.52 | 1.38  (0.44, 4.34) | 0.59 | 0.38  (0.08, 1.84) | 0.23 | 1.36  (0.43, 4.28) | 0.60 | 0.37  (0.08, 1.83) | 0.22 | 0.45  (0.13, 1.55) | 0.21 | 1.19  (0.29, 4.84) | 0.81 | 0.99  (0.38, 2.54) | 0.98 | 0.41  (0.13, 1.25) | 0.12 | 0.61  (0.27, 1.35) | 0.22 |
| Hypertension | 0.20  (0.03, 1.49) | 0.12 | 0.61  (0.20, 1.88) | 0.39 | 0.31  (0.07, 1.40) | 0.13 | 1.33  (0.51, 3.44) | 0.56 | 1.04  (0.29, 3.70) | 0.95 | 1.29  (0.61, 2.74) | 0.51 | 1.14  (0.32, 4.01) | 0.84 | 1.13  (0.54, 2.37) | 0.75 | 0.85  (0.32, 2.29) | 0.75 | 1.17  (0.65, 2.10) | 0.60 |
| Lung condition (any) | 0.42  (0.07, 2.64) | 0.36 | 1.54  (0.57, 4.19) | 0.40 | 0.47  (0.10, 2.26) | 0.35 | 1.13  (0.39, 3.22) | 0.83 | 0.69  (0.18, 2.60) | 0.59 | 1.17  (0.52, 2.66) | 0.70 | 1.29  (0.37, 4.50) | 0.69 | 1.04  (0.46, 2.34) | 0.92 | 0.90  (0.34, 2.44) | 0.84 | 1.10  (0.58, 2.10) | 0.77 |
| Mental health condition (any) | - | - | - | - | 0.40  (0.06, 2.79) | 0.36 | 1.41  (0.30, 6.59) | 0.66 | 0.80  (0.17, 3.69) | 0.77 | 1.73  (0.54, 5.58) | 0.36 | 4.08  (1.07, 15.6) | **0.040** | 6.52  (2.40, 17.7) | **<0.001** | 1.41  (0.39, 5.07) | 0.60 | 2.85  (1.08, 7.53) | **0.034** |
| Number of comorbidities | 2.64  (0.65, 10.7) | 0.18 | 1.02  (0.66, 1.56) | 0.95 | 1.93  (0.91, 4.09) | 0.09 | 1.43  (0.99, 2.05) | 0.05 | 1.38  (0.63, 3.03) | 0.42 | 1.06  (0.77, 1.48) | 0.71 | 1.02  (0.49, 2.13) | 0.97 | 1.16  (0.85, 1.57) | 0.35 | 1.26  (0.70, 2.29) | 0.45 | 1.11  (0.86, 1.43) | 0.41 |
| **Admission data** | | | | | | | | | | | | | | | | | | | | |
| Number of acute COVID-19 symptoms | 0.95  (0.71, 1.27) | 0.73 | 0.97  (0.76, 1.25) | 0.83 | 0.99  (0.74, 1.31) | 0.93 | 1.04  (0.81, 1.33) | 0.75 | 0.67  (0.51, 0.87) | **0.003** | 0.79  (0.63, 0.99) | **0.038** | 1.05  (0.85, 1.31) | 0.66 | 1.15  (0.95, 1.37) | 0.15 | 0.91  (0.76, 1.08) | 0.28 | 1.02  (0.88, 1.19) | 0.78 |
| Time from onset of symptoms to time of follow-up appointment (days) | 1.01  (1.00, 1.03) | 0.16 | 1.01  (0.99, 1.02) | 0.39 | 1.01  (0.99, 1.02) | 0.51 | 1.00  (0.98, 1.01) | 0.56 | 0.99  (0.98, 1.01) | 0.30 | 0.99  (0.98, 1.00) | 0.17 | 0.99  (0.98, 1.01) | 0.46 | 0.99  (0.97, 1.00) | 0.06 | 1.00  (0.99, 1.01) | 0.75 | 1.01  (1.00, 1.02) | 0.07 |

*BMI: Body mass index; CFS: Clinical frailty scale (measured by Rockwood Frailty Scale). *Cerebrovascular disease and immunosuppression were excluded as predictors. *Table data are presented as adjusted odds ratio (aOR) and its 95% confidence ratio (95%CI).*
